# ‘Hidden’ work and lost opportunities: nursing research and impact case studies submitted to REF2021

**DOI:** 10.1177/17449871241261971

**Published:** 2024-10-06

**Authors:** Bridie Kent, Parveen Ali, Barbara Farquharson, Ruth Harris, Bridget Johnston, Daniel Kelly, Hugh Mckenna, Ann McMahon, Rachel M Taylor, Michael Traynor

**Affiliations:** Professor, University of Plymouth, Plymouth, UK; Professor, University of Sheffield, Sheffield, UK;; Doncaster and Bassetlaw Teaching Hospitals, Doncaster, UK; Associate Professor, University of Stirling, Stirling, UK; Professor, King’s College London, London, UK; Professor, University of Glasgow, Glasgow, UK; Professor, School of Healthcare Sciences, University of Cardiff, Cardiff, UK; Professor, Ulster University, Belfast, UK; Co-Editor in Chief, Journal of Research in Nursing; Honorary Professor, Plymouth University, Plymouth, UK; Professor, University College London Hospitals NHS Foundation Trust, London, UK; Independent Scholar and Writer about UK Nursing, London, UK

**Keywords:** clinical research, longitudinal research, research impact, research policy, workforce and employment

## Abstract

**Aim(s):**

(1) Identify and characterise the nursing contribution to impact case studies submitted to Research Excellence Framework (REF) 2021 across all Units of Assessment and compare this to those submitted to REF 2014; (2) Identify and characterise those impact case studies of relevance to nursing that did not include a nurse in the research team; (3) Compare the characteristics of impact case studies identified in Aim 2 with those that did include a nurse in the research team.

**Design:**

Desk-based analysis of REF2021 published data.

**Methods:**

We searched the REF2021 impact database with the term nurs* then sorted case studies into categories representing the involvement of nurses on the research team. We developed variables with which to examine the impact case studies (ICSs) and make comparisons.

**Results:**

One-fifth of ICSs involving a nurse researcher do not contribute to a visible body of ‘nursing research’ and is ‘hidden’ in other disciplines; research teams persistently fail to involve nurse researchers when researching topics of clear relevance to nurses and nursing.

**Conclusion:**

Our findings provide insight into two topics of importance for nursing research: reputation, and failure to benefit from transdisciplinarity.

**Impact:**

Benefit to nurse researchers from involvement in transdisciplinary research is still limited; some nursing research remains hidden.

## Introduction

An occupational group that wants to claim professional status needs, among other characteristics, to be able to demonstrate a significant knowledge base for its practice (Abbott, 1988; Davies, 1995; Jamous and Peloille, 1970; Walby and Greenwell, 1994). In contemporary societies, this involves an association with universities. Universities are considered to be not only the site for the education of students, and hence a gatekeeper to the professions, but also a place for the generation of the knowledge that is imparted, in other words, research. In the early 1990s nursing in the United Kingdom made a significant move forward in professional status when its students moved into higher education institutions (United Kingdom Central Council for Nursing Midwifery and Health Visiting, 1986). And as long ago as the 1970s an influential report into the profession recommended that nursing become a research-based profession (Committee on Nursing, 1972). Considerable efforts to fulfil this ambition have been expended since then, and research by nurses into problems of relevance to the profession has developed considerably, with a number of centres of excellence now established along with multidisciplinary work (Rafferty et al., 2001; Traynor et al., 2001). Nevertheless, across the university sector as a whole, nursing struggles to be involved in high prestige, highly funded research and to build lasting research capacity. In many universities, nursing departments are primarily focussed on the delivery of degree level education, the numbers of staff with higher research degrees is relatively small and what research is carried out can be piecemeal and parochial.

### The REF and its predecessors

One formal measure of research quality that has been in operation in the United Kingdom since 1986, is the government’s regular assessment of university research activity, currently known as the Research Excellence Framework (REF). The REF and its predecessors assess research on a disciplinary basis. In 2008, the research landscape was assessed under 67 discipline-based ‘units of assessment’, and this was reduced to 36 in 2014 and 34 in 2021 in an attempt to reduce complexity and cost. There was a discrete unit of assessment for nursing from 1986, but in 2014 it was subsumed into a unit with other practice-based health disciplines: allied health professions, dentistry and pharmacy, Unit of Assessment (UoA) 3. This research assessment provides an insight into the state of ‘nursing research’ carried out in universities, often though not always, in collaboration with partners within the UK National Health Service (NHS). Because of the competitive nature of the exercise and the ‘league tables’ that it generates, nursing as a research discipline is visible for comparison against other disciplines, though, since 2014, those interested specifically in nursing research need to disaggregate its performance from the other disciplines involved in UoA3. This is not always straightforward and this disaggregation, along with an analysis of the result, is what is offered in this paper. Second to the significant research funding that flows to universities from the government as a result of REF (£2Bn), reputation is probably the most important outcome. Universities select the most flattering REF metrics with which to describe their various research centres in recruitment and other publicity. However, for the reasons outlined above, the REF results are important also to whole disciplines, such as nursing, which are concerned with their ongoing research profile and reputation. An upbeat commentary on nursing research as ‘coming of age’, and the profession *as a whole* moving away from an identity as ‘medicine’s poor relation’ as a result of the 2008 assessment (Lipsett, 2008) bears witness to the importance of the REF and its predecessors. This reputational advance was particularly welcome as the discipline sat uncomfortably at the bottom of the disciplinary league table in previous years (Lindsay et al., 2003). Despite this good news for the professions’ reputation, research excellence is still not spread evenly across the sector. Although 124 UK universities offer nursing degrees and courses, only 90 universities made entries into the REF2021 UoA3, and some of those entries did not involve nursing departments. This reveals that not every university with a nursing department felt it advantageous to submit its research into this UoA. In fact the relatively impressive performance of nursing in 2008 was attributed, in part, to the decision of many nursing departments, or whoever made the decision on their behalf, *not* to enter the exercise. Robinson and colleagues claimed that entries from ‘too many people and too many departments’ had ‘undoubtedly contributed towards our bottom position’ (Robinson et al., 2002). So being entered into REF or not is a crucial factor not only for individual departments but for the discipline as a whole (Thompson and McKenna, 2022), and is worthy of study.

### Hidden contribution and lost opportunity

Researchers of nursing’s involvement in previous assessment exercises have explored the issue of research undertaken by nurse researchers being submitted to units of assessment other than nursing (Lindsay et al., 2003; Traynor and Rafferty, 1998a). Prominent among the historic reasons for this, according to a survey of leaders of nursing departments that were not entered into the nursing UoA, was a belief that there was insufficient quantity or quality to merit an entry (Traynor and Rafferty, 1998b). So, if we wish to capture and characterise the entirety of university research carried out by nurses or with a nurse as a member of the research team we need to locate these pieces of work in whichever UoA they are included, beyond UoA3 (given that some, possibly a great deal of, research will not be included in REF at all). Such research, that is, university research by nurses not entered into the REF’s UoA3, has been considered as the ‘hidden’ contribution of nurses to the research endeavour (Kelly et al., 2016). Related to this is a second area of complexity. An examination of the entire output of the REF reveals research on various aspects of nursing work, for example patient interventions delivered by nurses, that has not been entered into UoA3, nor does there appear to be an individual with a nursing background identified as a member of the research team. We will argue that this type of work represents a lost opportunity on the part of the research teams that developed research on a topic of close relevance to nurses but did not take advantage of the knowledge that a nurse researcher might have brought to the development, conduct and implementation of the research. Nurse researchers as a whole also fail to gain the benefit that involvement in such teams might bring. Related to the topic of implementation, we now go on to discuss research impact and its measurement within research assessment.

### Impact

In 2014, the REF included for the first time an attempt to measure the ‘impact’ of research beyond the university. Impact has been defined as ‘an effect on, change or benefit to the economy, society, culture, public policy or services, health, the environment or quality of life, beyond academia’ (McLellan, 2020). The most recent REF, undertaken in 2021, was the second to include impact case studies. The aim of including impact studies was to provide some visible accountability for the considerable public investment in research and to produce evidence of the benefits of this investment to the UK Treasury. Higher Education Institutions entering the REF are required to submit impact case studies (ICSs), arguing for and giving evidence of the benefit to wider society of their research work, focussed on particular research projects or programmes. Health and well-being benefits figure as a significant category of societal impact and because of this, research studies of relevance to nursing work are likely to be seen as good candidates for such ICSs. While a great deal of research of relevance to nursing will not be included in university REF entries, or be the subject of an impact case study, we suggest that those research projects that have been selected by universities preparing for this highly competitive exercise could be considered as among the most high-quality and impactful research and so worthy of study.

Analysis of those ICSs carried out by nurses or with nurses as members of the research team can reveal important information about the priority concerns of the profession, that is, research topics, as well as frequently used methodologies, multidisciplinary partners, geographical spread, sources and levels of research funding and other characteristics. The detailed and publicly available REF data make such an analysis possible. And because REF2021 was the second appearance of impact case studies, a comparison across this 7-year period is a way of identifying changes in the characteristics of ICSs with relevance to nursing. Given the caveats above, that is, that not all research done by nurses will be included in the REF or in REF impact studies, this analysis can provide a rare overview of recent research done by nurses and possible changes over time. A working definition of what can be considered research ‘of relevance to nursing’ is offered below. In addition, an analysis of REF ICSs across all units of assessment can reveal the extent of the ‘lost opportunity’ created by research of strong relevance to nurses proceeding without the contribution of a nurse in the research team. Having identified such ‘lost opportunity’ ICSs, a comparison between this group of case studies with those that do include a nurse may go some way to reveal the character of the contribution of nurse researchers to the research endeavour.

## Aims of the study

1. Identify and characterise the totality of the nursing contribution to ICSs submitted to REF 2021 across all UoAs and compare this to those submitted to REF 2014.
2. Identify and characterise those ICSs of relevance to nursing but that did not include a nurse in the research team.
3. Compare the characteristics of ICSs identified in Aim 2 with those ICSs that did include a nurse in the research team.

## Method

We replicated our 2014 approach to analysis of the ICSs (Kelly et al., 2016), but collected additional data. Our first step was to identify any ICS submitted in 2021 that involved nursing researchers or was done on topics of relevance to nurses. Because of the anticipated problem of ‘hidden’ research, that is, ICSs not submitted to UoA3 where nursing is located, we searched the entire database of ICSs submitted to REF. This database is available from https://results2021.ref.ac.uk/impact. We used nurs* as our single search term and searched in December 2022. We used this single broad term because it would be the most inclusive for the search with the understanding that it may identify a number of ‘false positive’ results, that is, ICSs with no relevance to nursing.

Our second step was to identify and exclude ICSs of no relevance to nursing, and to categorise each remaining ICS retrieved in relation to nurse involvement in the research team, that is, ascertain whether a nurse was identified among the research team or not. We developed three categories: Category 1 indicated research concerned with the practice or on a topic of relevance to nursing and was undertaken by a team containing at least one nurse, ‘research undertaken by nurses’; Category 2 comprised research on the practice or another aspect of nursing, but where nurse inclusion in the research team was not apparent, ‘research on nurses or nursing’; Category 3 comprised ICSs where the research had no direct or immediate relevance to nursing but may have been relevant in a more generic sense to health and social care. This last category could be considered as comprising the ‘false positives’ mentioned above and was not included in our subsequent analysis.

All case studies identified in the initial search were assessed independently by two reviewers and allocated to one of the three categories. Detailed internet searches were undertaken, where needed, to determine the discipline and profession of the researchers named in a case study as this was not always stated. Each pair of reviewers cross-checked at least 10% of the ICSs between them, discussed any differences and came to an agreement about each. Meetings of the whole team took place at three points in the process to further cross-check, discuss and refine categorisations. Nevertheless, categorisation of a study as of relevance to nursing was a result of the judgement of the reviewers, albeit with processes aimed at improving rigour built in.

As in 2014, examples of ICSs from each category were first identified and then extracted from the REF database (see Supplemental Material).

In addition to categorising each ICS, the research team extracted a range of data: information about the topic, method/s, funding and funding sources, professional background of the researchers, the type and reach of impact claimed, the institutions where the research was conducted and whether any outputs cited in the case studies were published in nursing journals, in total some 22 variables. Not all of these are presented in this paper. This work was completed by April 2023. Results were compiled into an Excel spreadsheet, and this was imported into SPSS for analysis.

### Ethical considerations

This study comprised an analysis of material made public by the UK funding councils on the website that provides the details of all of the entries for REF2021, including the names of all researchers entered. Because of this existing public availability, we considered that the study did not raise any ethical issues.

## Findings

Using nurs* as a search term, 576 case study entries were initially retrieved from the REF database, representing 8.5% of the total 6781 case studies submitted to REF in 2021. This is a slightly higher proportion than in 2014 when 469 of 6975 (7%) of case studies were found using the same method. The remainder of this analysis includes only those case studies considered to fit into categories 1 and 2, that is, those with a clear relevance to nursing. Where a noteworthy difference between the characteristics of category 1 and category 2 studies is apparent, we present a table indicating this. We do this to address aim 3, to explore whether studies that declared the involvement of nurses in their research team might tend to be different in any way from those that did not. In order to address aim 1, we compared our data from 2014 with the present study, where it was available.

The number of case studies allocated into categories 1 and 2 in 2021 and 2014 are shown in Table 1.

**Table 1.** Category of impact case study.

Category	2021 *n* = 181	2014^a^ *n* = 130	χ^2^	Sig.
Category 1: Research undertaken by nurses	111 (61.3)	80 (61.5)	0.001	.970
Category 2: Research on nurses or nursing	70 (38.7)	50 (38.5)		

*n* (%).

a Kelly et al. (2016).

Of these case studies, 109 were submitted into UoA3 and the next highest number, 12, were submitted into UoA4, Psychology, Psychiatry and Neuroscience. Although the number of included case studies is larger in 2021 than 2014, the proportions of studies in the two categories are similar.

In order to address aims 1 and 3, we compared the sources of funding for the retrieved studies across categories 1 and 2. Table 2 shows the result for all studies and compares categories 1 and 2.

**Table 2.** Sources of funding by Category and totals REF2021.

Funding body	All *n* (%)	Category 1	Category 2
BEIS Research Councils, The Royal Society, British Academy and The Royal Society of Edinburgh	16 (8.8)	7 (6.3)	9 (12.9)
EU government bodies	10 (5.5)	9 (8.1)	1 (1.4)
EU-based charities (open competitive process)	1 (0.6)	0	1 (1.4)
UK central government bodies/local authorities, health and hospital authorities	71 (39.2)	48 (43.2)	23 (32.9)
UK industry, commerce and public corporations	6 (3.3)	2 (1.8)	4 (5.7)
UK other sources	4 (2.2)	2 (1.8)	2 (2.9)
UK-based charities (open competitive process)	22 (12.2)	15 (13.5)	7 (10)
UK-based charities (other)	14 (7.7)	5 (4.5)	9 (12.9)
Not stated/not clear	37 (20.4)	22 (19.8)	15 (21.4)

BEIS: Department for Business, Energy & Industrial Strategy; EU: European Union; UK: United Kingdom.

The largest group of the category 1 and 2 ICSs received funding from UK central government bodies/local authorities or health and hospital authorities (*n* = 71; 39%). Most funding came from UK sources with only a small number of studies being funded by EU bodies (*n* = 11; 6%). If we compare funders across category 1 and 2 studies, the only major difference is that 8.1% of category 1 studies received EU government funding compared with 1.4% of category 2. Actual numbers, however, are small. It is notable that one-fifth of case studies did not include information about funding sources.

We recorded the amount of funding declared in the case studies. The most frequent category of funding was less than £250k (Table 3).

**Table 3.** Amount of funding REF 2021.

Amount of funding	Category 1 *n* = 111	Category 2 *n* = 70
<£250k	26 (23.4)	20 (28.6)
⩾£250 and <£500k	14 (12.6)	6 (8.6)
⩾£500k and <£1M	11 (9.9)	4 (5.7)
⩾£1M and <£5M	32 (28.8)	11 (15.7)
⩾£5M and <£10M	5 (4.5)	4 (5.7)
⩾£10M	1 (0.9)	0
Not stated	22 (19.8)	25 (35.7)

*n* (%).

Category 1: Research undertaken by nurses; Category 2: Research on nurses or nursing.

A quarter of case studies overall did not state the amount of funding received. Studies with a nurse in the team, and those without, have a slightly different pattern of funding with a tendency for the former to report higher levels of research funding (42% of studies ⩾£500k had a nurse on the team vs 27% in studies without that level of funding stated [(χ^2^, 5.29, *p* < 0.05]).

In order to understand the priority research topics of university nurse researchers, we devised topic categories for the case studies in 2014 and used these for the present analysis in order to identify any changes. The outcome is reported in Table 4. In 2014, topic data were only collected for category 1 studies, so we have separated category 1 and 2 studies for the 2021 data to enable comparison.

**Table 4.** Topic of impact case study.

Topic of Impact Case Study	Category 1 – 2021 *n* = 111	Category 1 – 2014*^a^ *N* = 80	χ^2^	Sig.	Category 2 – 2021 *n* = 70
Patient safety	18 (16.2)	19 (23.8)	1.685	.194	12 (17.1)
Policy and practice evaluation	17 (15.3)	17 (21)	1.119	.290	19 (27.1)
Reproductive/women’s health	11 (9.9)	10 (12.5)	0.319	.572	7 (10)
Quality of life	20 (18)	10 (12.5)	1.069	.301	10 (14.3)
Mental health	6 (5.4)	9 (11.3)	2.195	.138	5 (7.1)
Death and dying	4 (3.6)	8 (10)	3.231	.072	5 (7.1)
Workforce	17 (15.3)	5 (6.3)	3.749	.053	7 (10)
Other	18 (16.2)	2 (2.5)	9.330	.002	5 (7.1)

*n* (%).

* Category 1 only reported in 2014; ^a^Kelly et al. (2016).

Considering category 1 studies only, that is, those concerning research undertaken by nurses, we can see an increase in the number and proportion of case studies focussing on workforce and quality of life although the difference is not statistically significant. There was a significant increase in topics falling into the ‘other’ category in 2021. All other topics show fewer or a similar number of ICSs in 2021.

Most authors of ICSs identified the research designs used in their underpinning work, or, where they did not, the design/s used were often apparent from the titles of research publications listed. Table 5 shows the number of times, in category 1 and 2 studies, that designs were used as well as case studies where there was no information about research design. Many ICS authors identified a number of discrete studies as contributing to impact, often with their own designs, for example, a systematic review followed by a clinical trial.

**Table 5.** Research designs used in the case studies.*.

Research Design	*n* (%)
Qualitative	82 (23.4)
Other	59 (16.9)
Systematic review	46 (13.1)
RCT	43 (12.3)
Observational/cohort	41 (11.7)
Survey	32 (9.1)
Not clear/not stated	25 (7.1)
Other intervention	22 (6.3)

* Total = 350 due to multiple studies and methods used in some case studies.

The most frequently adopted design was described as ‘qualitative’. Often no further details about the specific qualitative approach were provided. Broadly similar proportions of category 1 and 2 studies were associated with the various research designs, although of the 82 instances of qualitative studies, 56 of these were from category 1 studies, that is, those involving a nurse. The clarity with which information on research design was provided was poor, making detailed comparison between categories 1 and 2 difficult.

Finally, we examined the location of the research publications cited to underpin the ICSs. We did this in order to determine whether the presence of a nurse on the research team was associated with a different publication profile, that is, including publications from specifically nursing, midwifery or health visiting journals. Here, a clear difference between category 1 – research teams with a nurse – and category 2 – those without – was apparent (see Table 6). Teams with a nurse were much more likely to include publications in nursing journals. (47% vs 13% (χ^2^: 22.19, *p* < 0.001)

**Table 6.** Research outputs in ‘nursing’ journals.

Published in ‘nursing’ journals	Category 1 *n* = 111	Category 2 *n* = 70
Yes	52 (46.8)	9 (12.9)
No	58 (52.3)	61 (87.1)
Not clear/not stated	1 (0.9)	0

*n* (%).

Category 1: Research undertaken by nurses; Category 2: Research on nurses or nursing.

## Discussion

### Our findings provide insight into two topics of importance for nursing research:

#### 1. Reputation

This is the first study to seek to identify the full contribution of nurse researchers beyond that submitted to UoA3 to the United Kingdom’s national assessment of research quality and impact. Research studies entered into REFs (both as publications and as ICSs), and, of course, resultant high ratings, can add to the reputation of individual research centres as well as whole disciplines (Thompson and McKenna, 2022). This is related to the broader question of the possible reputation-enhancing work of a particular discipline being entered into REF at all.

We identified 111 ICSs featuring research that included a nurse on the research team. Of these, 22 were submitted to units of assessment other than UoA3. This proportion represents the level of ‘hidden’ research done by nurses, leaving 89 or 80% of these case studies submitted to UoA3. Nurses’ research work was ‘hidden’ largely in clinical medicine, public health, psychology and social work and social policy, disciplines that might be considered cognate to nursing. As a long series of research and opinion articles have claimed, nursing’s level of visibility in REF, as well as its performance, can add to its reputation as a research-based profession and to its reputation within universities (Lipsett, 2008; Lindsay et al., 2003; Robinson et al., 2002; Traynor and Rafferty, 1999; Thompson and McKenna, 2022). Our findings suggest that 20% of this visibility and performance has been ‘hidden’ in REF2021.

Many UK universities have nursing departments or schools but not all of them make a nursing entry into REF. If nursing research capacity is small in any particular department, university decision-makers working towards maximising their REF outcome may instead enter individual high performing ‘nurse researchers’ into other related disciplines such as Social Policy and Social Work. This can contribute to a vicious cycle where a nursing department with no visible research profile is unlikely to attract aspiring nurse researchers in recruitment. University staff place high esteem on research activity as it is seen as a route towards promotion or even a requirement for job security. It is possible that nursing departments that are viewed as more or less ‘teaching only’ will have lower status within their universities.

There were a small number of apparent changes to the profile of the ICSs between 2014 and 2021. In terms of topic area, compared to 2014, a smaller proportion of category 1 case studies looked at patient safety and death and dying while there was a growth in the proportion of studies focussing on workforce and quality of life (see Supplemental Material for examples of studies). It would be hazardous to read from this any actual changes in priority of topic across nursing research as a whole as there are many reasons for particular case studies being selected for entry, however the increase in the proportion of studies on workforce may reflect the recent and current policy focus on problems of recruitment and retention and organisation of the nursing workforce (Buchan et al., 2020; NHS England, 2023). As previously mentioned, the relative proportion of category 1 to category 2 studies remained constant since 2014, that is, with 38% of all ICSs relevant to nursing not including a nurse. This consistent apparent failure to involve a nurse in such studies may be for many reasons, nevertheless it is disappointing in terms of the influence of nurse researchers and of the promotion of multidisciplinarity including nurses. This brings us to our second theme.

#### 2. Lost opportunity for impact

We have identified many research ICSs with apparent high relevance for nursing work or for the profession or organisation of nursing that do not appear to have involved any researcher with a nursing background in the research team. This accounts for just under 40% of all ICSs we identified. Of the 70 ICSs on nursing topics but which did not include a nurse on the team, a smaller proportion than category 1 studies, 30%, were submitted to UoA3. These case studies emerged from a very wide range of UoAs, including seven in clinical medicine or public health, seven in psychology and the remainder in a range of arts and humanities subjects. While of course not every piece of research with impact on the nursing aspect of NHS patient care will have been included in REF or as an ICS, where they have been included without the contribution of a nurse, it could be argued that this is a lost opportunity on the part of the research teams to benefit from the insight of a nurse when designing and implementing the study and planning its impact because of nursing’s patient-facing role. In addition, such an absence can make access to the profession, for example in order to introduce a change in practice, more difficult. In a part of the REF that focusses on various aspects of public benefit, it is all the more surprising that nearly 40% of ICSs with relevance for nursing did not include a nurse researcher. We might suggest that this is also a failure in the context of the widespread recognition of the importance of transdisciplinarity in research.

We found a range of different characteristics between category 1 and category 2 ICSs. Although differences in the funding source and amounts of funding were small, ICSs with a nurse reported the highest levels of research funding and a higher proportion of work with European funding. The reasons for this are not clear and the numbers are small, but one possible speculation might be that nurse researchers are relatively effective at gaining research funding. In terms of topic area, teams with a nurse conducted a greater proportion of research on ‘workforce’ issues and ‘quality of life’ and less on policy and practice evaluation. Again, the reasons for this are unclear though nursing workforce issues are clearly a current policy priority, as mentioned above. The profile of supporting publications was one area of clear difference with a little under half of the teams with a nurse including a publication from a nursing journal compared to just over 10% of teams without a nurse. This seems to represent an aim on the part of some of the teams with a nurse to reach a nursing audience with the findings of their research as part of their planned impact.

### Limitations

Not all research undertaken by nurses or of relevance to nursing work is entered into the REF, and if it is entered into REF may not be included among submitted ICSs; hence, there is likely to be more ‘impact’ on patients of nursing research than is included in the scope of this study. However, this study focusses on one important source of publicly visible research by nurses and ICSs entered into REF are likely to comprise exemplary research.

It was not possible to exactly compare research impact assessment between 2014 and 2021 because a number of changes were made to the detail of the REF between these two exercises. This included changes to the required number of ICSs submitted relative to the number of staff submitted to the exercise, hence, there were less ICSs submitted overall in 2021 than in 2014.

A methodological limitation was that the categorisation of ICSs was based on the judgement of the authors, albeit involving procedures aimed at minimising the effect of purely subjective judgements. The team undertaking the categorisation changed between 2014 and 2021 and this could be a source of inconsistency.

## Conclusion

The results of the United Kingdom’s REF provide unique insights into the character of university-based research carried out by nurse researchers. Our work has revealed two characteristics that are important for the nursing research enterprise as a whole. These are that approximately one-fifth of university research carried out involving a nurse researcher does not contribute to a visible body of ‘nursing research’ and is ‘hidden’ in the research outputs of other disciplines. In addition, research teams persistently fail to involve nurse researchers when researching topics of clear relevance to nurses and nursing. This results not only in a lost opportunity for those teams but also in a failure of nurse researchers to benefit from involvement in potentially high-quality research work and teams. University based research in nursing has made significant progress since its early low rankings. There is, however, still progress to be made if its potential is to be achieved.

### Key points for policy, practice and/or research

- Approximately one-fifth of university research carried out involving a nurse researcher does not contribute to a visible body of ‘nursing research’ and is ‘hidden’ in the research outputs of other disciplines.
- Research teams persistently fail to involve nurse researchers when researching topics of clear relevance to the nursing profession.
- Nursing’s level of visibility in the REF can be improved further; a number of universities chose not to submit an entry as ‘nursing’ in UoA3.

## Supplemental Material

sj-pdf-1-jrn-10.1177_17449871241261971 – Supplemental material for ‘Hidden’ work and lost opportunities: nursing research and impact case studies submitted to REF2021 Supplemental material, sj-pdf-1-jrn-10.1177_17449871241261971 for ‘Hidden’ work and lost opportunities: nursing research and impact case studies submitted to REF2021 by Bridie Kent, Parveen Ali, Barbara Farquharson, Ruth Harris, Bridget Johnston, Daniel Kelly, Hugh Mckenna, Ann McMahon, Rachel M Taylor and Michael Traynor in Journal of Research in Nursing
