# Supplementary material for: ‘Hidden’ work and lost opportunities: nursing research and impact case studies submitted to REF2021
Source: J Res Nurs. 2024 Oct 6;29(6):469–80. doi: 10.1177/17449871241261971 (PMC11539177; doi:10.1177/17449871241261971)
Supplement: sj-pdf-1-jrn-10.1177_17449871241261971 – Supplemental material for ‘Hidden’ work and lost opportunities: nursing research and impact case studies submitted to REF2021 [file sj-pdf-1-jrn-10.1177_17449871241261971.pdf]

Supplementary material

Table S1. Example Impact case studies from Categories 1 and 2 by topic heading

Patient safety

Category 1

Saving lives by improving structured responses to sepsis patient deterioration

The University of the West of Scotland UoA3

https://results2021.ref.ac.uk/impact/451f9a1a-fd4e-4d6b-af53-7be8cf12dcde?page=1

Mismanaged patient deterioration is a leading cause of safety-related death. Sepsis, a leading cause of deterioration, kills 11 million per annum, globally. Prior to our research, early warning scores, used to improve detection and responses, were only used in hospitals. We validated these scores for both adult and children in the prehospital setting, in order to facilitate early recognition of patient deterioration. These scores are now used across the UK and internationally. Our research also informed on how to deliver a sepsis treatment bundle, including which monitors to use. State-of-the-art approaches established through our research have been pivotal to practice changes driven by the Scottish Patient Safety Programme. In Scotland alone, our research has contributed to a 21% reduction in Sepsis mortality and a 31% reduction in cardiac arrests.

Category 2

Inclusive design for healthcare: creating impact in clinical practice and commercial investment by addressing barriers to innovation

The Royal College of Art UoA 32

https://results2021.ref.ac.uk/impact/ea55c991-8326-4680-90dc-6d08530228ed?page=1

RCA research led by Jonathan West and a team in the Helen Hamlyn Centre for Design expanded the use of inclusive design techniques to address procurement and regulatory factors that constrain innovation in hospital and community health design. Building on the RCA's participatory design approach with patients and frontline staff, the work resulted in significant impact in *clinical practice* (10,000 'Wee Wheel' pocket guides produced by Public Health Wales enabling nurses to assess urine output easily and accurately); in *clinical trials*(362 people with paranoia in three NHS areas participated in the 'SlowMo' digital platform clinical trial); and in *commercial development* (the 'Flomark' redesign of the hospital drip has raised over £400,000 from investors). This research also enabled the HHCD team to work

with hospitals and Public Health Wales to address challenges during the Covid-19 pandemic.

Policy and Practice evaluation

Category 1

Using a theory of person-centredness to transform nursing and healthcare cultures

Queen Margaret University, Edinburgh UoA3

https://results2021.ref.ac.uk/impact/a6ef7c3a-e83a-4346-a1b6-df5721f78cd5?page=1

Led by nursing academics at Queen Margaret University, Edinburgh, the theoretical frameworks, implementation methods and evaluation processes have influenced national developments in nursing and healthcare in 22 countries to increase quality of patient experience and staff well-being and improve workplace culture e.g., a national programme of development entitled the Enhanced Practice Experience for Service Users in the Republic of Ireland, the 'Essentials of Care' Programme developed by the NSW Australia State Health Department and a model of nursing home accreditation in Austria.

Category 2

Novel decision support modelling to increase efficiency of healthcare provision, reduce costs and improve patient experience

University of Hertfordshire UoA17

https://results2021.ref.ac.uk/impact/850ec906-6199-44a4-9fef-8e364db8fda1?page=1

This research developed decision support systems to increase efficiency of a variety services, improve patient care, reduce waiting times, increase compliance with treatment time targets and costs savings. In particular, the decision support system enabled the Ophthalmology Department of an NHS Trust to forecast future service demand for regular intravitreal injections for patients with diabetic macular oedema in its retinal service. Several scenarios which would enable the Trust to fulfil increased demand in a cost-effective way were explored and as a result the Trust recruited 2 nurse injectors. This resulted in decreased waiting lists and led to setting up specialist nurse posts to manage follow-up appointments.

Reproduction/Women's health

Category 1

Influencing Change in UNICEF's Baby Friendly Hospital Initiative in the UK and World-wide

University of Central Lancashire UoA3

https://results2021.ref.ac.uk/impact/a2dd9225-0968-41ea-af8f-4499ec63c718?page=1

The research conducted by the Maternal and Infant Nutrition and Nurture Group (MAINN) has created UK and worldwide impact through their influence on the WHO/UNICEF Baby Friendly Hospital Initiative (BFHI), the global initiative to optimise infant feeding practices. The research has been highly influential, ensuring that UNICEF's embrace of bonding and relationship development between parent and infant is at the centre of infant feeding care. The group's research is extensively cited in the UNICEF UK's supporting evidence publication, leading to changes in the standards of the UNICEF UK Baby Friendly Initiative, staff education, university education, assessment processes and associated resources. This paradigm shift towards relationships is now being replicated in over 150 countries, delivering positive impact on breastfeeding rates and contributing toward an associated impact in nine Sustainable Development Goals.

Category 2

Breaking the menopause taboo at work: changing the lives of people experiencing menopause in employment

University of Leicester UoA17

https://results2021.ref.ac.uk/impact/745f1828-f627-4a07-a012-59e11434ebd0?page=1

Led by business/economist, the findings and recommendations from this report catalysed media and national interest in supporting employees experiencing menopause symptoms in the workplace. The public and legal discourse and subsequent policy and service development influenced by the publication of [R2] have led to a transformation in the way in which issues relating to the menopause are managed at work, reported in the media and recognised in society. The direct impacts include workplace menopause policies across a spectrum of employers in the UK [E5] and the development of toolkits and resources by professional bodies and national and public organizations such as the Chartered Institute of Personnel and Development, the Civil Service, the Higher Education Academy, and the NHS.

Quality of Life

Category 1

The Carer Support Needs Assessment Tool intervention (CSNAT-I): enabling comprehensive, tailored support for family carers

University of Cambridge UoA4

https://results2021.ref.ac.uk/impact/051a5032-fc8b-4615-81f7-434dd8d8a07f?page=1

At any given time more than half a million people in the UK are providing care to someone who is in their last year of life, with significant effects on all aspects of carer health and well-being. The Carer Support Needs Assessment Tool intervention (CSNAT-I) is a comprehensive, person-centred approach for supporting family carers which meet the need for evidence-based practice in end-of-life care (EOLC). The UK Royal College of General Practitioners (RCGP) and health departments in Australia and Norway recommend CSNAT-I. Training in its use has been delivered face to face to 134 UK organisations, and to teams in eight countries. Development of an online CSNAT Approach Training and Implementation Toolkit now enables practitioners nationally and internationally to implement CSNAT-I. As of July 2020, 168 organisations in 12 countries are licensed to use CSNAT as a practice intervention, and another 73 research licences have been issued for 58 organisations to use in 26 countries. The tool has been translated into 15 languages, allowing delivery of targeted, tailored support to carers worldwide

Category 2

Mother-infant proximity in the postnatal period

University of Durham UoA22

https://results2021.ref.ac.uk/impact/074dbbe5-bc19-47b8-a1b1-c04c74624b69?page=1

We demonstrated the benefits of using side-car cribs in postpartum settings and the close link between increased breastfeeding frequency and mother-baby night-time contact via three randomised trials of night-time care on a United Kingdom postnatal ward. Our findings led to changes in recommended best practice in postnatal care in the United Kingdom and overseas. We showed that the use of stand-alone bassinettes to accommodate babies in mothers' room impeded interaction between mothers and babies at night, leading to poorer breastfeeding initiation, unsafe handling, and low maternal confidence. Consequently, United Kingdom hospitals began using 3-sided cribs with an open side adjacent to the mother's bed on postnatal wards. United Kingdom and international organisations have also used our findings from the trials to develop new policies for staff and guidance for parents emphasising the importance of mother-baby night-time contact for breastfeeding initiation and continuation. In the United Kingdom our work affects the postnatal care of 630,000 mothers and babies annually. Since 2002 the Durham University Anthropology Department's Infancy & Sleep Centre (formerly the Parent-Infant Sleep Lab) team has researched the implications of mother-infant proximity during postnatal hospitalisation on breastfeeding and infant safety outcomes using a commercial bassinet known as a 'clip-on' or a 'side-car' crib **[R1-R5].** This 3-sided bassinet with an open side is positioned adjacent to the mother's bed and secured in place to provide a continuous infant sleep-surface with no barrier between mother and baby.

With the support of a Consultant Neonatologist overseeing the postnatal ward we conducted a series of three randomised controlled trials (RCT), with qualitative follow-up, at a tertiary-level hospital.

Mental Health

Category 1

Safewards: Increasing safety on psychiatric inpatient wards

King's College London. UoA4

https://results2021.ref.ac.uk/impact/f6f22422-62a6-4335-87a1-7507431a3971?page=1

King's Safewards Model proposes 6 domains which can give rise to flashpoints triggering conflict and/or containment. These domains were: the staff team, the physical environment, factors outside hospital, the patient community, patient characteristics and the regulatory framework. The model identified that staff interventions can modify these processes, reducing conflict and the need for containment, and is unique in its recognition that care staff's use of containment, even when motivated by the desire to prevent future conflict, can cause further conflict to occur. King's described this dynamic model systematically and showed how understanding its central principles can inform strategies that promote the safety of patients and staff, leading to a healthier work and recovery environment.

Category 2

Changing museum practice to create cultural legacies for mental health groups and other communities

University of Glasgow UoA14

https://results2021.ref.ac.uk/impact/b7473081-3f08-4dec-9802-1d4afd0b5649?page=1

McGeachan's research into a unique 'arts and mental health' collection, *Art Extraordinary* (AE), fostered a new model of academic-museum curatorial practice, enacted and evaluated across Glasgow since 2015 in partnership with Glasgow Museums (GM) and GM's Open Museums (OM). This work produced innovative community co-curation of AE in public exhibitions, with evidence showing a direct increase in the skills, confidence and empowerment of 290 participants from mental health and community groups. Two impact legacies emerge: (1) McGeachan's successful model of academic-museum curatorial practice levered new GM-OM investment in an 'Art Outside the Box' Handling Kit, to reach an estimated 25,000 people annually, institutionalising the benefits of the partnership work; (2) McGeachan's research and model of partnership working secured a new GM commitment to allocate *permanent* exhibition space representing AE and mental ill-health in the flagship Kelvingrove Museum, which receives an estimated 1.3 million visits annually. Together, these impacts have changed GM's engagement with mental-ill health.

Death and dying

Category 1

Enhancing and expanding the scope, reach and value of volunteers in palliative and end-of-life care

University of Lancaster Unit 3 A

https://results2021.ref.ac.uk/impact/f847d204-d75e-4179-8715-81a05bb7c484?page=1

The case study focuses on enhancing and expanding the scope, reach and value of volunteers in palliative and end-of-life care. Lancaster research has improved the provision of palliative care in two distinct ways: by increasing the scale and reach of volunteers in palliative and end-of-life care through evidence-based practice, and by forming a high-quality research base that has actively been used for UK-based and European policy around volunteer-provided palliative care. The case study included four key areas of research.

Lancaster research was the first to establish the scale and scope of direct care volunteering within UK hospices (between 2011 and 2013) that that included a national survey of hospices and palliative care services, extensive literature reviews, and in-depth case studies. The research team demonstrated the scale of volunteering that was in existence. The Lancaster team also established an evidence base for the potential value of volunteer-provided care was established with a randomised controlled trial of volunteer-provided end-of-life care. The research team also targeted the need for scaling up and ensuring longevity of a wider range of evidence-based volunteer roles. Finally they developed an evidenced based tool kit to guide the future development and provision of end-of-life care volunteering services.

Category 2

Increasing Public and Professional Understanding of Death, Dying and Palliative Care through Film

Queen Mary University London: UoA 33

https://results2021.ref.ac.uk/impact/15b5a2db-1250-40e6-8751-2691eb841034?page=1

A practice led film by the team at Queen Mary University London concerned with terminal illness and the dying process, has advanced professional and public understanding about tend of life and palliative care. They achieved this through the development and implementation of educational and clinical training materials in correspondence with the production and dissemination of two interrelated film works: ISLAND, a feature-length film released theatrically in the UK; and The Interval and the Instant, a multiscreen gallery video installation which they toured internationally. They developed the films with palliative care clinicians and patients. The film artworks both depict and interrogate terminal illness and the dying process. They

have been disseminated, with an accompanying educational toolkit, in the fields of palliative care and medicine, partnering with both Hospices and NHS Trusts. They piloted the film and toolkit with 150 trainee nurses and doctors The toolkit is currently also being licensed by NHS Trusts and Hospices for staff and student training in end-of-life care, as well as to international university libraries.

They argue the film makes death and dying accessible to a general audience, and to professionals and medical students within the field of palliative care. They argue the film reaches diverse audiences and creates opportunities for engagement and familiarity with a challenging subject. Through UK and international screenings, exhibitions and targeted talks and workshops, They used of innovative contexts to give unprecedented visibility to palliative care and to the end of life.

Workforce

Category 1

Influencing changes to nurse staffing legislation, policy and practice to improve patient safety in hospitals in the UK and internationally

University of SouthamptonUoA3

https://results2021.ref.ac.uk/impact/90d5f685-40d8-423e-b30b-cb946bed66aa?page=1

Research at the University of Southampton has influenced legislation, policies and campaigns to create safer staffing on hospital wards around the world, by exposing the patient safety risks of low nurse staffing levels. Errors and omissions in care are common and lead to thousands of avoidable deaths; our research showed that low nurse staffing and skill mix are factors in many of these deaths. The findings motivated change by providing core evidence for: global policy (World Health Organisation, International Council of Nursing); legislation (Wales, Scotland); changes in practice (Staffing Framework in Ireland); safe-staffing campaigns (e.g. Royal College of Nursing, Safe Staffing Alliance); and the development of safe-staffing guidelines in the UK (NHS Improvement, and National Institute for Heath and Care Excellence – NICE). Increases in nurse staffing in line with the 2014 NICE guidelines, are estimated to lead to 1,760 fewer deaths per year in English hospitals.

Category 2

Transforming healthcare leadership and organisational cultures to deliver high-quality, compassionate care

University of Lancaster (Business school)

https://results2021.ref.ac.uk/impact/fa03de70-0d8f-4e15-b37a-9bc2fb81fb55?page=1

A collaborative research project led by West investigating the structural and cultural factors that threaten the safety and quality of care throughout the NHS has underpinned systemic impact within the healthcare systems throughout the UK. The research has been a catalyst for the transformation of leadership and cultures across the sector, becoming central to policy embedded in national strategies such as: ‘Developing People, Improving Care’ (2016) and ‘We Are the NHS: The People Plan for 2020/2021’. The partnership between West and NHS England and NHS Improvement has supported NHS Trusts in England through a programme to develop leadership and cultures that deliver high-quality, compassionate patient care, reaching half a million clinical and managerial staff. Similar approaches have been developed in Wales, Northern Ireland and Scotland. West was appointed a CBE in the Queen’s 2020 Birthday Honours List for services to compassion and innovation in the NHS. The other key members of the team and their universities were Dawson (Sheffield), Baker, Dixon-Woods, Martin (Leicester), McKee (Aberdeen), Lilford (Birmingham), and Wilkie (National Association for Patient Participation).

‘Other’

Impacts in the ‘other’ category included a disparate range of topics including preventative healthcare, housing, sexual and child health. We sub-categorised 4 ICSs as concerning ‘healthcare education’; 3 concerned child health; 3 cancer, and 2 concerned preventive healthcare.

Category1

Integrating health, care services and housing: innovative and improved ways of helping older people

University of Northumbria at Newcastle UoA3

https://results2021.ref.ac.uk/impact/828c27b8-99d3-49a9-be6c-f0618a3206d9?page=1

Public services must meet the challenge of supporting older people who live with diseases and disabilities to continue living independently for as long as possible. One way to do this is to integrate health and care services with housing. Northumbria University researchers have focussed on these integration processes and identified that, to be successful, services needed to identify and respond to issues early, upskill housing staff, and create pathways for integrated working across services. This research led to three key impacts. 1) New models of integrated services for older people were created that have resulted in reduced hospital admissions in North Tyneside and enable older people to live in their own homes for longer, thereby reducing the cost of care. 2) The approach has led to a collaboration with master planners and policy makers to shape the design of housing developments that aim to support independent ageing-in-place. 3) The research was used by The Centre for Ageing Better to lobby the UK Government for better funding of the Disabled Facilities Grant (funding home adaptations), helping drive subsequent budget increases of GBP92,000,000 between 2018-2020, taking annual funding to GBP505,000,000.

Category 2

Improving Breast Cancer Outcomes in Older Patients

https://results2021.ref.ac.uk/impact/8b6c42d4-b20f-487d-bc28-e90fe4843807?page=1

Sheffield Hallam University UoA3

Older women with breast cancer have poorer outcomes than younger women, partly due to the non-standard treatment they receive. As there is little to guide older patients or clinicians when faced with a treatment choice, decisions are often based on personal preference, rather than being evidence-based. Sheffield Hallam research developed a decision support intervention (DESI) to guide optimal, personalised treatment for older women with certain breast cancers. This led to: changes in clinical practice and treatment received, improved patient knowledge, greater shared decision-making, enhanced quality of life and a sense of empowerment. The DESI has benefitted both clinicians and older women with breast cancer. The DESI quickly achieved global reach, having been used by clinicians on all six continents.
